# Impairments of Speech Comprehension in Patients with Tinnitus—A Review

**DOI:** 10.3389/fnagi.2017.00224

**Published:** 2017-07-11

**Authors:** Daniela Ivansic, Orlando Guntinas-Lichius, Boris Müller, Gerd F. Volk, Gerlind Schneider, Christian Dobel

**Affiliations:** Tinnitus-Centre, Department of Otorhinolaryngology, Jena University Hospital Jena, Germany

**Keywords:** tinnitus, speech comprehension, speech processing, cognition, review, aging

## Abstract

Tinnitus describes the subjective perception of a sound despite the absence of external stimulation. Being a sensory symptom the majority of studies focusses on the auditory pathway. In the recent years, a series of studies suggested a crucial involvement of the limbic system in the manifestation and development of chronic tinnitus. Regarding cognitive symptoms, several reviews addressed the presence of cognitive impairments in tinnitus as well and concluded that attention and memory processes are affected. Despite the importance for social communication and the reliance on a highly functional auditory system, speech comprehension remains a largely neglected field in tinnitus research. This is why we review here the existing literature on speech and language functions in tinnitus patients. Reviewed studies suggest that speech comprehension is impaired in patients with tinnitus, especially in the presence of competing noise. This is even the case in tinnitus patients with normal hearing thresholds. Additionally, speech comprehension measures seem independent of other measures such as tinnitus severity and perceived tinnitus loudness. According to the majority of authors, the speech comprehension difficulties arise as a result of central processes or dysfunctional neuroplasticity.

## Introduction

### Impairment of General Cognitive Functions in Tinnitus

Tinnitus describes the perception of a sound in the absence of physical stimulation and is also called a phantom percept. As tinnitus is characterized as a sensory and auditory phenomenon, most studies addressed dysfunctions of the auditory pathways. Several studies suggested that not only peripheral, but also central auditory systems and dysfunctional neuroplastic processes as a consequence of deafferentation contribute to the development of the impairment (Møller, 2007). Consequently, therapeutic approaches focused on the auditory system (e.g., Jastreboff et al., 1996; Eysel-Gosepath et al., 2004; Stein et al., 2016). However, research provided evidence that not only auditory pathways are affected, but also the limbic system which is involved in affective processing (see e.g., Husain, 2016). The support for this was not only provided by imaging studies, but was also gained from clinical reports stressing the high comorbidity of tinnitus with psychiatric disorders (Andersson, 2002).

### Reviews on Cognitive Functions

Due to neuroplasticity and the strong linkage of auditory pathways, the limbic system and central processes, it is not surprising that a range of cognitive processes are impaired in tinnitus patients. To date, there are three reviews with each having a slightly different focus (Andersson and McKenna, 2006; Mohamad et al., 2016; Tegg-Quinn et al., 2016).

In their review, Andersson and McKenna (2006) grouped studies in three areas: (1) neuropsychological measures evidenced that control of attention is impaired under conditions when attentional demands are neither high nor low, but average; (2) regarding cognitive biases, selective attention bias was described as an emphasis on disorder-related information (e.g., found in patients suffering from anxiety disorders), while memory bias has been found among depressive patients, who tend to remember negative information better than positive. Such biases were present in tinnitus patients as well, but there may be two subgroups of tinnitus patients with one exhibiting a focus on tinnitus related information (similar to anxiety disorders) and the other displaying memory distortions as seen in depression; and (3) finally, Andersson and McKenna (2006) report the literature on the conscious appraisal of tinnitus (i.e., in terms of remembering and reporting the intrusiveness of tinnitus) and they stress the prominent role of complaints, worries and anxious thoughts.

In a systematic literature search on tinnitus and cognition with a focus on attention and memory, Mohamad et al. (2016) reviewed nine studies. While there is mixed evidence for the notion that working memory and selective attention are affected in tinnitus patients, a clearer picture evolved for executive attention. This function encompasses the engagement and disengagement to a specific stimulus, as well as the switch to a different stimulus to reach a specific goal. These processes enhance processing of relevant stimuli and in turn achieve a better representation of information in working memory. For instance, tinnitus patients were generally slower than controls in Stroop tasks (Andersson et al., 2000; Jackson et al., 2014) and also exhibited a deficit in an “attention network test” which is used as an index measure for executive control (Heeren et al., 2014). Additionally, self-reported tinnitus symptoms correlated with behavioral measures for executive control (Heeren et al., 2014; Jackson et al., 2014). Reviewing this evidence, Mohamad et al. (2016) concluded that there is at least preliminary evidence that tinnitus interferes with executive attention.

Recently Tegg-Quinn et al. (2016) performed a similar systematic review, however, with a broader search criterion and a focus on clinical management of invasive tinnitus. They report 18 studies employing a variety of measures on cognitive functions in tinnitus. Based on nine studies reporting attentional impairments, the authors state that executive control of attention is impaired and impacts on cognitive function in tinnitus patients.

Apart from these reviews, Roberts et al. (2015) also stress the crucial involvement of attentional processes in the generation of chronic tinnitus and provide a model for these dysfunctional processes. In normal hearing persons, neural patterns from actual inputs are compared to predicted representations generated from long-term memory. If the factual and the predicted inputs match, the comparison leads to a cancellation and no mismatch signal is generated. In tinnitus patients, cochlear damage and/or subsequent neuroplastic processes lead to a mismatch between the two inputs and the comparison process draws attention towards the auditory input to generate a better representation of the acoustic environment.

Taken together, several reviews agree that tinnitus is accompanied by cognitive impairments particularly affecting attentional executive functions. Given the reliance of speech comprehension on detailed and stable representations of the acoustic environment, it seems surprising that speech is not a more dominant topic regarding cognitive impairments. This is even more so, when considering the tight connections between attention and language. We will briefly describe these processes before we review the studies on speech, language and tinnitus.

### Language Comprehension, Production, Working Memory and Attention

There is a strong link between language and attention at several levels and a comprehensive overview would go far beyond the scope of the current review. The best example for this link is the role of joint attention during language acquisition when infants want that their communication partners devote their attention to the same object in space as they do (Moore and Dunham, 1995). Similarly, speakers generally look at objects before they name them and so the order of naming becomes evident from the order of looking (e.g., Meyer and Dobel, 2004; Dobel et al., 2011) and listeners look at objects that are mentioned by a speaker (e.g., Henderson et al., 2003). Besides this overt behavior, there are earlier states of processing when attention plays an important role. The connection between language comprehension, working memory capacity (WMC) and attention is possibly best captured by the *Ease of Language Understanding* (ELU) model (for a recent overview of the model see Rönnberg et al., 2013). With regard to early encoding of auditory stimuli, the interplay of attention and WMC ensures a fine-tuning of processing even under adverse conditions. Several neurophysiological studies suggest that attentional processes interact with WMC already at subcortical levels (Zouridakis et al., 1998; Sörqvist et al., 2012; Tsuchida et al., 2012). Next to these early effects, the interplay of attention with WMC and their influence on short-term retention was evidenced by studies on persons wearing hearing aids, where short-term memory performance correlated with the degree of hearing impairment under conditions of divided attention (Tun et al., 2009; Rönnberg et al., 2011). Thus, on one hand, hearing aids reduce attentional costs when persons listen to speech in noise and can improve speech comprehension (Rönnberg et al., 2013). On the other hand, tinnitus is likely to put strong demands on speech processing in everyday listening situations, or more explicitly: “signal distortion will tax WMC during speech understanding” (Rönnberg et al., 2013; p. 10).

On this background, i.e., the presence of cognitive and particularly attentional deficits in tinnitus patients and the strong link between attention and speech, we predict that speech comprehension impairments might be a prominent symptom in patients with chronic tinnitus, especially under difficult listening conditions. Here, we want to substantiate this claim by a systematic review. Second, we want to find out if this impairment is caused by peripheral cochlear damage and is as such a rather trivial phenomenon. Finally, we would like to give some recommendations for future research based on our findings.

## Methods

### Search Strategy

**Figure 1:**
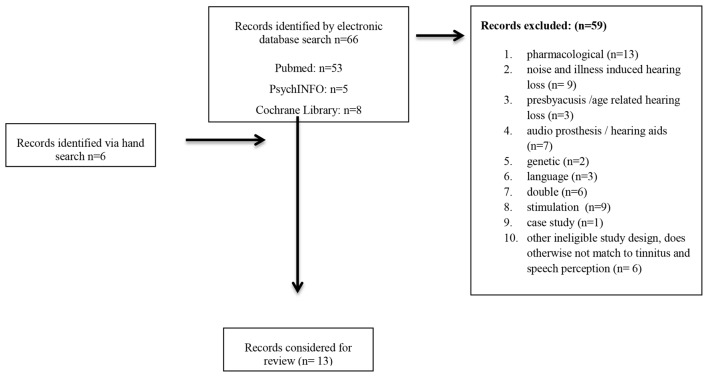
Flow chart for the identification of studies included in the review.

A literature search with no date restriction using the search terms (((tinnitus) AND (“speech perception” OR “speech recognition”)) NOT (implant* OR transplant*) NOT review NOT Schwannoma NOT cancer NOT sudden NOT Menière) was undertaken in the PubMed and PsycINFO databases as well as the Cochrane Library in September 2016. Additional articles were obtained through the references of studies identified during the initial search (see Figure 1). The search resulted in *N* = 13 publications reported below and summarized in Table 1.

**Table 1:** Overview of reviewed studies: basic participant characteristics, employed speech comprehension tests, main results and conclusions regarding our hypothesis language.

	Methods			Results		Conclusion
Study	Participants	Speech comprehension (SC) test(s)	Language	Impairment in SC for T	Impact of hearing condition	Involvement of central processes
Newman et al. (1994)	*N* = 23: UT + BHL *N* = 23: BHL	Harvard Psychoacoustic Laboratory word test, synthetic sentence identification test, speech perception in noise test, dichotic sentence identification test	English	yes	yes	yes
Goldstein and Shulman (1999)	*N* = 25: severe T + NH	Monaural low-pass filtered speech, binaural fusion test, rapid alternating speech test, competing sentence test, staggered spondaic word test	English	yes	yes	yes
Cuny et al. (2004)	*N* = 20: UT *N* = 10: BT *N* = 30: healthy controls	Dichotic listening, lateralized lexical decision	French	yes	n.a.	yes
Huang et al. (2007)	*N* = 20: T + NH	Mandarin speech perception in noise test	Mandarin	yes	yes	yes
Hennig et al. (2011)	*N* = 19: T + NH + hyperacusis *N* = 23: controls	Sentences recognition threshold in silence and in noise	Portuguese	yes	yes	n.s.
Paglialonga et al. (2011)	*N* = 10: NH	Speech in noise test with three-alternative, forced-choice paradigm	Italian	yes	yes	n.s.
Ryu et al. (2012)	*N* = 20: T + NH *N* = 20: healthy controls	Korean version of hearing in noise test	Korean	yes	no	n.s.
Soalheiro et al. (2012)	*N* = 495: T	Logoaudiometric thresholds without competing noise	Portuguese	yes	n.a.	n.s.
Mertens et al. (2013)	*N* = 15: UHL and ipsilateral T	Speech reception threshold using the Leuven intelligibility sentences test	Dutch	yes	n.a.	yes
Jain and Sahoo (2014)	*N* = 10: mild T *N* = 10: moderate T *N* = 20: controls	Speech perception in noise based on sentences from the Kannada quick speech in noise test	Canarese	yes	n.a.	yes
Moon et al. (2015)	*N* = 9: UT + NH *N* = 12: UT + HL *N* = 9: BT + HL *N* = 15: healthy controls	Speech recognition in noise	Korean	yes	n.a.	yes
Gilles et al. (2016)	*N* = 19: T *N* = 68: controls	Speech-in-noise testing with continuous and modulated noise based on the Leuven intelligibility sentence test	Dutch	yes	n.a.	yes
Tugumia et al. (2016)	*N* = 12: UT + BT	Speech-in-noise test (not specified)	Portuguese	no	n.a.	n.s.

*U, unilateral; B, bilateral; T, Tinnitus; HL, hearing loss; NH, normal hearing; SC, speech comprehension; n.a., not applicable; n.s, not stated*.

## Results

With regard to our hypothesis, we will briefly outline the overall results (for an overview see Table 1). From the 13 studies reported here, 12 evidenced impairments of speech perception in patients suffering from chronic tinnitus. Across studies a variety of tests was used with and without competing noise. Patients were native speakers of rather different languages. As the only exception, Tugumia et al. (2016) did not report impaired speech perception in tinnitus patients. Six studies contrasted speech comprehension under easy (e.g., no competing noise) and difficult conditions. Five of those reported stronger impairments under difficult conditions. In 8 of the 13 studies, the authors interpreted their results in terms of central contributions to the generation of speech comprehension impairments. In the remaining studies, the authors did not comment on this. In the following we will present the reviewed studies in more detail and focus on the hypotheses outlined in the “Introduction” Section.

Newman et al. (1994) investigated the relationship between psychoacoustic measures and speech perception abilities in patients with hearing loss and tinnitus (THL group) compared to patients with hearing loss only (HL group; *N* = 23 in each group). Even though peripheral hearing loss was similar in both groups, the THL group performed worse than the HL group in speech-in-noise tasks, particularly for difficult items. There was no correlation between speech perception abilities and perceived handicap. The authors suggested that tinnitus should be considered as a symptom which may also have central causes rather than only peripheral damage. Hearing loss does not even have to be present for the occurrence of impairment in speech perception as reported by Soalheiro et al. (2012). This study evidenced impaired speech perception measured without competing noise in 81% of almost 300 workers exposed to occupational stress.

Goldstein and Shulman (1999) investigated 25 individuals with severely disturbing tinnitus. About half of these participants (52%) displayed low scores in at least one of the central auditory speech tests. More specifically, in the low-pass filtered speech test and the competing sentence test, patients complaining about interference of tinnitus with daily communication displayed low performance. Similar to Newman et al. (1994), Goldstein and Shulman stressed the contribution of central processes to the generation of chronic tinnitus. Huang et al. (2007) investigated speech perception in 20 chronic tinnitus patients speaking Mandarin. Compared to controls, tinnitus patients displayed generally lower scores in perceiving sentences in noise and, as it appears (though it was not analyzed), this was more so when sentences were hard to predict, i.e., with low contextual cues. As above, these measures did not correlate with tinnitus loudness or disability, again arguing for a contribution of central processes to the symptomatology. A corroborating result for the influence of hearing condition was obtained with 19 Portuguese patients suffering from tinnitus and hyperacusis (Hennig et al., 2011). Compared to normal hearing controls they demonstrated lower abilities to comprehend sentences in noise, but performed normally without noise. Using the Korean version of the hearing in noise test, Ryu et al. (2012) showed that tinnitus patients with normal pure tone audiometry (*N* = 20), thus arguing for no peripheral impairment, performed worse than normal hearing controls, both when sentences were displayed in silence and with competing noise, i.e., there was no influence of hearing condition. Similar findings were reported on 15 Dutch speakers wearing cochlear implants suffering from one-sided tinnitus (Mertens et al., 2013). Importantly, this study evidenced impaired speech-in-noise comprehension in the unaffected ear which argues again for impairment with strong contributions from central processes (note that publications studying patients with hearing aids were excluded from this review; this study was included, however, because the unaffected ear was tested). Similarly arguing for an involvement of central processes, Cuny et al. (2004) reported left hemispheric dominance (i.e., a right ear advantage) in controls employing a word dichotic listening and lateralized decision task. In contrast, tinnitus patients did not show such a hemispheric asymmetry for language processing. Importantly, tinnitus was simulated in normal hearing participants by a tinnitus-like noise and thus the difference between groups could not be attributed to the tinnitus noise *per se* (note that the inclusion of white band noise worsens speech intelligibility even in normal hearing participants, Paglialonga et al., 2011). Consequently, the authors argued for functional reorganization as a consequence of the peripheral damage. The same conclusion was reached by Moon et al. (2015) who measured the speech recognition threshold in noise in 21 patients suffering from tinnitus. Patients performed worse than a control group in this test, but not in a variety on tests on auditory spectral and temporal resolution. This dissociation argues again against the hypothesis that tinnitus depends on damage to outer hair cells, but rather on plastic changes in the central auditory pathways after damage to the cochlea.

To further investigate the dissociation between inconspicuous pure tone audiograms and speech perception, Jain and Sahoo (2014) tested 20 patients suffering from mild to moderate tinnitus in comparison to a control group. Tests included speech perception in noise, frequency discrimination, differential limen of intensity and frequency, gap detection and modulation detection thresholds. The results demonstrated that tinnitus patients were impaired in various aspects of auditory perception such as frequency discrimination, temporal resolution and speech recognition in noise, i.e., aspects that are not reflected in a pure tone audiogram. Corroborating results were presented from 19 young adults exposed to leisure noise who developed noise-induced tinnitus (Gilles et al., 2016). While these participants did not show differences to a control group with regard to hearing thresholds, otoacoustic emissions and ABR, participants with noise induced tinnitus displayed worse abilities for sentence comprehension in noise. Thus, even if measures indicating peripheral damage are inconspicuous, speech perception tests argue for impairment.

## Discussion and Conclusion

Given the small but recently increasing literature on problems with speech perception in tinnitus, it appears that these functions are impaired in tinnitus patients across a wide variety of languages. Despite the small number of studies, the reported evidence for tinnitus-related impairments of speech perception goes far beyond just a few cases. From an experimental viewpoint, in most studies the investigated group sizes allowed to make robust conclusions (all *N* > 14). The only study that did not find evidence for speech comprehension impairments is by Tugumia et al. (2016). They used a Portuguese version of a speech-in-Noise test (Pereira and Schochat, 2011) as a validation of an auditory training procedure. Speech comprehension did not improve due to training and was already in the normal range before training. Reasons for this divergence to other studies might be the rather mild condition of the studied patients as well as their young age.

As a particularly important finding in several studies, speech measures were impaired even if audiological measures were inconspicuous (e.g., Ryu et al., 2012; Mertens et al., 2013; Gilles et al., 2016). Thus, a speech impairment was even demonstrated when no peripheral damage was evidenced. We take this as an argument that reduced speech comprehension is a phenomenon *per se* and not only attributable to impaired hearing. Consequently, it is not a trivial problem as one might have quite understandably suggested.

Nevertheless, the underlying mechanism is far from understood. Despite the strong link between attention and speech comprehension as well as language in general, it is not yet clear what the nature of their relation is. Are the impairments in speech processing a direct consequence of attentional impairments or are they both the consequence of underlying common functions? The majority of authors agree that for the development of chronic tinnitus peripheral damage is accompanied by neuroplastic processes of central functions (e.g., Newman et al., 1994; Cuny et al., 2004; Paglialonga et al., 2011; Mertens et al., 2013). If attentional functions and/or working memory and/or language processing are *per se* affected, then this should become obvious independent from the domain of input. This assumption is to some degree supported by the very high percentage of tinnitus patients reporting difficulties with concentration (Watts et al., 2016) and by evidence for poorer reading performance in these patients (Sanchez and Stephens, 1997). As a working hypothesis, we propose that difficulties in speech comprehension arise via a central mechanism. The phantom noise attracts attention and as a consequence these resources are not available for other processes. This becomes expressed as deficits of divided or selective attention. As described in the introduction and emphasized by the ELU model, language processing depends on all levels on attentional capacities and is consequently impaired. We outline below some possibilities to investigate this on a detailed functional level. As an alternative hypothesis, this mechanism could be mediated via stress responses to the tinnitus. Such a possibility seems plausible regarding the recent evidence for the involvement of the limbic system (e.g., Husain, 2016).

### Outlook

We propose that future research should aim at disentangling the various factors that contribute to impaired speech perception in tinnitus as well as the causal relationships between attention and speech comprehension. By comparing complex sentences (with e.g., relative clauses) to simpler sentences, the interaction with working memory can be tested. The use of text passages requiring frequent or rare shifts of attention could evidence the interplay of speech processing with attentional requirements. Eye-tracking proved as a useful technique to measure shifts and the locus of attention and could therefore be applied in tinnitus-research to investigate the interplay of speech processing and attentional focus (e.g., Andersson et al., 2011). Moreover, keeping in mind that different phonemes are plotted on different frequency areas in an audiogram, it would be interesting to investigate which phonemes are harder to understand than others. As an example on the single word level, it is highly plausible that words with fricatives are particularly hard to process, especially with competing white noise. While these are only a few suggestions, they necessitate the development of systematic, standardized test batteries employing speech stimuli involving not only tinnitus-researchers but linguists, phoneticians and neuroscientists alike. The multitude and variability of symptoms demand that tinnitus research becomes even more interdisciplinary than it already is.

## Author Contributions

DI, BM and CD: literature search, conception of review; wrote the manuscript. OG-L, GFV and GS: conception of review; wrote the manuscript.

## Conflict of Interest Statement

The authors declare that the research was conducted in the absence of any commercial or financial relationships that could be construed as a potential conflict of interest.
